# Notes on the Value of Cocaine in Minor Surgery

**Published:** 1887-10

**Authors:** A. P. Morgan Vance


### NOTES ON THE VALUE OF COCAINE IN MINOR SURGERY.

By A. P. Morgan, Vance, M. D.

Since the advent of cocaine as a local anæsthetic, I have used it in all cases of minor surgery where it was at all possible. The marked success in all instances prompts me to give some clinical data showing its great value in many operations that formerly required general anæsthesia, *e. g.*, the removal of inflamed toe nails, amputation of fingers or toes, any of the different tenotomies, searching for needles, removal of superficial tumors, circumcision, opening of felons, palmar abscesses, bubos, removal of sequestra from the tarsus, removal of extensive epulis, ligation of arteries, neurectomy, etc.

I have so far met with no accidents, one case only having any disagreeable symptoms. The drug was used hypodermatically in nearly all cases, the anæsthesia being continued by topical applications into the wound, when this was opened, the 4-per cent. solution being used subcutaneously. For topical use the 6 or 8-per cent. solution was used.

The genuineness of the drug must be unquestionable, if good results are expected. The only failures I have had could be traced to the doubtful character of the drug used.

I can in no better way illustrate its action than by the relation of some of the cases:

CASE I was a neurectomy of the musculo-cutaneous nerve on the dorsum of foot, in the case of a very nervous young lady. An Esmarck bandage being first applied, ten drops of the 4-per cent. solution was injected under the skin in the line of the nerve. In four minutes the incision, one inch in length, was made without pain, the lady looking at the procedure. The only difficulty in the operation was the locating of the nerve filament, as there was complete numbing of the nerve—the sensation, which is the usual guide in the operation, being totally absent. Finally, what was thought to be the nerve was removed, the result showing this supposition correct.

I have seen it questioned whether cocaine will act when injected into inflamed tissues. The following case will be of interest upon this point:

CASE II.—Patient of Dr. W. T. Leachman, with very painful and excessively inflamed bubo. The woman was very nervous, and full of dread of the pain of incision. Five minutes after the injection of 15 drops of the 4-per cent. solution, a free incision was made, while the patient was conversing with Dr. Leachman, it being some minutes afterwards before she knew of the use of the knife.

CASE III.—Division of shortened faseia in the palm of a gentleman's hand, which was contracted, drawing the ring-finger into marked flexion. An injection of 10 drops of cocaine solution was given. Four minutes afterward, a complete subcutaneous division of the hand was made in two places, with the patient remarking that it hurt him no more than if the knife had been stuck in the door-jamb.

CASE IV.—Removal of two epitheliomas from an old man's face; one with a circumference of nine inches, the other of four; the first after six injections of 10 drops of the 4-per cent. solution, the second after two injections, the whole of the surfaces being curetted after the topical application of an 8-per cent. solution. The patient complained of no pain, but said he felt the pulling of the spoon during the scraping. There was no alarming constitutional effect, though the old man became very talkative, and appeared tipsy, whether from an ounce of whisky administered or from the cocaine, I am unable to say.

CASE V.—An operation upon the glans penis for the relief of hypospadias, in a boy of ten years. This is the only case where I was disturbed by the symptoms produced. Five minutes after the injection of 6 drops of the 4-per cent. (?) solution into the gland, the boy remarked in a sleepy way that he was going to sleep. The operation was proceeded with, a tenotome being passed through the gland from the false meatus to the opening behind. Just at this point, the patient was observed to be very quiet, as if sleeping very profoundly. Upon efforts to arouse him, he was discovered to be in a state of apparently complete coma. His lips were pale, heart very feeble, and his whole condition alarming. He was revived in a few minutes, however, by slapping his face with a wet towel, and by shaking him sharply, and completely relieved upon swallowing $\frac{1}{2}$ ounce of whisky. He said: "I just could not keep awake." No bad result followed. This patient had no pain. Upon investigation of the specimen of cocaine used in this case, the scales upon which the drug was weighed were found very defective, and I believe the solution was much stronger than 4-per cent., hence the symptoms.

CASE VI, Dr. F. C. Leber.—Acute stage of Pott's disease of the spine, where two deep eschars were made with the large button of a Pacquelin cautery. After two injections of 4-per cent. solution of cocaine, one on either side of the kyphos, with no pain whatever being felt by the patient, a lady of 46 years of age.

CASE VII.—Young lady with an acquired talipes equinus, in whom the tendo achillis and the five superficial extensor tendons of the toes were divided subcutaneously, after the injection of cocaine, with no pain; 5 drops used on either side of the tendo achillis, and 4 drops used with each extensor tendon, five minutes time being allowed between the injections and the use of the knife.

I have done the various tenotomies for talipes many times in the past two years, both in adults and children, without complaint on the part of the patients; also tenotomies elsewhere, *e.g.*, of the hamstring tendons in false ankylosis, and division of the tendons of the sterno-cleido-mastoideus for torticollis.

CASE VIII.—Young lady with two-thirds of large milliner's needle, for two weeks in the palm of her hand. With the use of an Esmarck bandage, and injection of cocaine, a bloodless and painless search, with considerable dissection was made, and the foreign body was removed from the deeper structures. I don't know any more satisfactory class of cases for the employment of the drug than those of which the above is an example.

CASE IX.—Amputation of a ring-finger at metacarpo-phalangeal articulation. The patient was an old man, who was nervous from long suffering, and dreaded the operation very much. Two injections of 15 drops each were introduced on either side of the joint, the Esmarck bandage having been previously applied. There was no pain complained of in this case, though twenty minutes elapsed before the stitches were introduced.

CASE X.—Was the removal of an extensive epulis from the upper jaw of a lady patient of Dr. F. C. Wilson. The tumor was very large; all the teeth except the wisdom teeth had been extracted long before. The alveolar process was greatly hypertrophied, being many times greater than natural, and there were polypoid masses of the growth that bridged over the roof of the mouth, almost filling it. The fibrous covering of the process was very dense, so much so, that it was difficult to introduce a needle into it. We feared the employment of chloroform or ether in this case, on account of the great danger of hæmorrhage, into the larynx and trachæ, especially as the greatest difficulty had been experienced on a former occasion, when the lower jaw was operated on for the removal of a similar growth, and subsequently at an attempted removal of the present growth. Consequently, we decided to attempt the operation under the local influence of cocaine.

The lady had nerved herself to go through with the operation come what might. I began by injecting at several points a 4-per cent. solution, expecting to be able to remove the fibrous tissue only. After removing considerable of this, I determined that to remove this alone would be of little service, as the bony base was so much enlarged and incorporated with the overlying fibrous tissues. The patient having had no pain so far, I removed a piece of the alveolar, with no complaint, and I continued literally paring down the whole process with a knife, until sufficient was removed to insure a good foundation for the artificial teeth, the anæsthesia being kept up ahead of the knife, by the application topically, and as far as possible by injection. After the work upon the bony process was finished, the polypoid growth in the roof of the mouth was scraped off with a sharp spoon, and the actual cautery applied thoroughly to the surface. The operation lasted nearly four hours, and the patient suffered so little that the complaints were trifling, the extraction of the two teeth causing more pain than all the rest of the work.

Twenty-nine grains of the solution was used; of course, only a small part of this was absorbed. Whisky was given freely during the operation, the only disagreeable symptom being a little dizziness upon attempting to walk.

CASE XI.—The removal of an ivory exostosis from the temporal region of a young gentleman, the growth probably, being excited by a blow. It was about as large at its base as a silver half dime, projecting above the surface a quarter of an inch. An injection of 4-per cent. solution was made over the mass, 10 drops being introduced, an incision three-fourths of an inch long being made down to the bone without pain, the mass of bone being removed with gouge, forceps, and chisel, the only complaint being of the jar when the mallet was used. The wound healed by immediate union.

When one thinks that none of the operations above mentioned could have been done formerly without general anæsthesia, *the value* of cocaine in minor surgery can be appreciated.

I will state, in conclusion, that I have seen no untoward results, in the way of increased reaction, preventing early union in wounds following the use of cocaine. I have seen no difference in this respect from what would be expected under other circumstances without the use of cocaine.—*Southwestern Medical Gazette.*

